# Effect of Climate Change on Food Industry Supply Chain Resilience in China on the Basis of Double Machine Learning Models

**DOI:** 10.3390/foods14213623

**Published:** 2025-10-24

**Authors:** Shengtian Jin, Dongxian Liu, Luchen Huang

**Affiliations:** 1 School of Finance, Anhui University of Finance & Economics, Bengbu 233000, China; 2 Institute of Statistics and Applied Mathematics, Anhui University of Finance & Economics, Bengbu 233000, China; 3 College of Economics & Management, Northwest A&F University, 3 Taicheng Rd., Yangling District, Xi’an 712100, China; 4 Department of Agricultural Economics, Kiel University, 24118 Kiel, Germany; 5 Sino-German Center for Agricultural and Food Economics, 3 Taicheng Rd., Yangling District, Xianyang 712100, China

**Keywords:** climate change, food industry supply chain resilience, double machine learning, crop diversification

## Abstract

In recent years, global climate fluctuation has been obvious and has had a significant impact on the food industry system, which makes the impact of climate change on the resilience of the food industry supply chain of great concern. Based on this, this paper selects the panel data of 30 provinces in China from 2011 to 2022; it takes the relationship between climate change and the toughness of the food industry supply chain as the entry point, and probes deeply into the intrinsic mechanism of the impact of climate change on the toughness of the food industry supply chain. The study found the following: First, climate change has a significant negative impact on the food industry supply chain resilience, and in climate change, the impact of temperature on the food industry supply chain resilience is significantly higher than the impact of rainfall on the food industry supply chain resilience. Second, the mechanism of the effect of climate change on food industry supply chains exhibits substantial heterogeneity between major food-producing regions and non-major food-producing ones and varies across different levels of mechanization. Third, crop diversification within the study scope remarkably mitigates the negative effect of temperature fluctuations on the resilience of the food industry supply chain. Therefore, the food supply chain system must enhance its capacity to withstand climate change, and current and future resilience should be strengthened by advancing the implementation of adaptation policies, plans, and actions that drive transformation.

## 1. Introduction

Food is the foremost priority of governance, and food security underpins national stability. Food security is the primary task for national development, and the report of the 20th Communist Party of China National Congress emphasizes the importance of comprehensively strengthening the foundation of food security [1]. As a major agricultural nation and a region that is sensitive to and substantially affected by global climate change, China faces unprecedented challenges. Currently, the world is undergoing a once-in-a-century transformation. Factors such as intensifying resource and environmental constraints, insufficient technological innovation, frequent extreme weather events, rising trade protectionism, and sudden public health emergencies have converged to severely disrupt global food industry supply chains [2]. This disruption has increased uncertainty and instability within food systems, posing a serious threat to China’s food security. The National Adaptation Strategy 2035 proposes fundamental principles: proactive adaptation with prevention as the priority, scientific adaptation in harmony with nature, systematic adaptation with focus on key areas, and coordinated adaptation with collaborative governance. It sets targets for 2035, including enhancing climate change monitoring and early warning capabilities, improving climate risk management and prevention systems, effectively controlling major climate-related disaster risks, establishing robust technical and standard systems for climate adaptation, elevating society-wide adaptive capacity, and building a climate-resilient society [3]. Climate change has substantially influenced global food production, with particularly pronounced effects on China, a major agricultural nation. Since 1961, the patterns and outcomes of climate change’s influence on China’s food production have been eliciting extensive attention. Research indicates that climate change affects food crop growth and development, planting structures, and pest and disease outbreaks through multiple pathways, thereby influencing food yield and quality. Rising temperatures shorten crop growing seasons, impairing plant development [4,5,6]. Enhancing the resilience of the food industry supply chain has become a critical pathway for comprehensively strengthening the foundation of food security and has major strategic value for effectively addressing climate risks and safeguarding national food security. Therefore, in-depth exploration of how climate change affects the resilience of the food industry supply chain is urgently needed.

Industry chains and supply chains fall under the domains of economics and management, respectively. While differing in perspective and content, they form an interconnected chain-network structure: supply chains serve as the microfoundation of industry chains, and industry chains constitute multiple supply chains. Together, they embody the chain-like interconnections driven by value creation within industrial division of labor [7,8]. Compared with regional resilience, industry supply chain resilience places greater emphasis on the connectivity attributes of network nodes [9]. Existing research on industrial and supply chain resilience has focused on the manufacturing sector and devoted limited attention to the food industry. As a special commodity crucial to the national economy and people’s livelihood, the resilience of the food industry supply chain possesses notable public attributes and foundational importance [10]. Existing studies generally agree that the resilience of food industry supply chains encompasses resistance capacity, adaptation capacity, and innovation capacity [11]. Enhancing the resilience of food industry supply chains concerns the stability of food supply and is crucial for ensuring national food security and promoting agricultural modernization. Through empirical research, some scholars have constructed evaluation indicator systems to measure the resilience of food industry supply chains [11,12] and explored their spatiotemporal evolution characteristics [13]. Other scholars examined the factors that affect resilience and found that agricultural technology [14], digital economy [15], technological innovation [12], inflation [16], and other factors exert remarkable effects on resilience. These studies revealed potential pathways for enhancing resilience from diverse perspectives, thus providing a theoretical foundation for relevant policy formulation.

**Figure 1:**
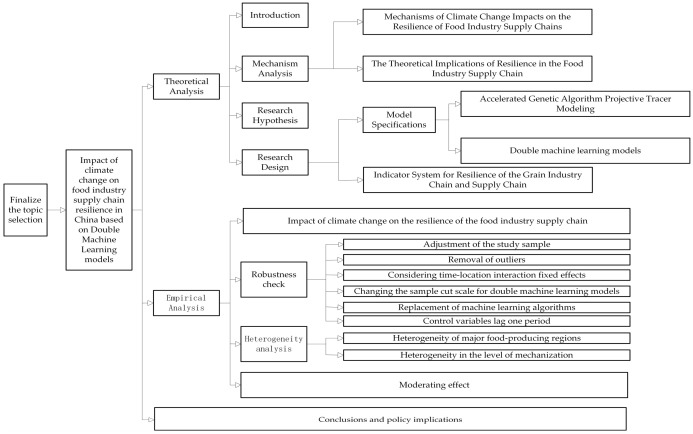
Article Concept Map.

In summary, existing research on the resilience of the food industry supply chain has focused on its construction and the influence of economic factors. Although climate change is also a notable influencing factor, research in this area remains limited. This study analyzes the topic from the following perspectives (Figure 1). First, it constructs an evaluation index system for the resilience level of the food industry supply chain on the basis of three capability dimensions. Second, it employs an accelerated genetic algorithm projection pursuit model to assess the resilience level of the food industry supply chain across 30 provinces in China from 2011 to 2022. Third, it constructs a double machine learning model to empirically analyze the effect of climate change on the resilience of the food industry supply chain while quantifying regional heterogeneity within this system. This work provides policymakers a relevant reference for formulating strategies aimed at enhancing the resilience of the food industry supply chain. Fourth, this research incorporates crop diversity into the dual machine learning model to explore whether crop diversification plays a moderating role in the process by which climate change affects the resilience of the food industry supply chain.

## 2. Materials and Methods

### 2.1. Theoretical Implications of Food Industry Supply Chain Resilience

The food industry serves as a vital cornerstone for high-quality development of the national economy and a key component of the real economy. Its systemic resilience holds decisive value for economic stability and public welfare. The resilience of the food industry supply chain refers to the system’s capacity to swiftly resume normal operations after encountering external shocks, ensuring timely access to nutritious and healthy food for all citizens while safeguarding the economic interests of all participants within the supply chain. This concept aligns closely with the core definition of China’s food system resilience, which emphasizes balancing the interests of all stakeholders while ensuring supply security. In practice, resilience manifests not only in the capacity to withstand shocks but also in the ability to recover swiftly after shocks occur; it involves long-term adaptability and transformative capabilities achieved through structural optimization and technological innovation. Specifically, grounded on core resilience theory established by Christopher and Peck [17] and on subsequent research that deepens the understanding of resource allocation and dynamic adaptation, resilience is deconstructed into three interconnected core dimensions: resistance capacity, innovation capacity, and adaptation capacity. The resilience of the food industry supply chain must prioritize addressing the transmission effects of external shocks, such as the influence of environmental factors (including climate change and natural disasters) on various segments of the supply chain. Only by enhancing the supply chain’s capacity to withstand disturbances and its post-disaster recovery capabilities can the stability of the food system be fundamentally safeguarded. This logic aligns perfectly with the research approach of building resilience in the food industry supply chain under environmental shocks, providing a theoretical convergence point for subsequent exploration of the specific effects of environmental factors on the resilience of the food industry supply chain as well as corresponding response strategies [18,19]. The 2024 China Agricultural Industry Development Report indicates that the dietary and consumption habits of Chinese residents are changing, and an increasing number of consumers are choosing convenient, healthy, and diversified food, which brings opportunities for the development of China’s agricultural processing industry. Therefore, in the future, we should optimize the regional layout of the agro-processing industry, promote the agro-processing industry’s countryside agglomeration, and drive the development of the countryside. Moreover, we should strengthen the support for scientific and technological innovation; encourage scientific research institutes, colleges, universities, and dragon-head enterprises to jointly build innovation platforms; improve the relevant qualified standard system; develop new productivity; cultivate new kinetic energy; and help enhance countryside re-industrialization. With reference to the definition of food industry supply chain resilience by existing scholars, the concept of the resilience of the food industry supply chain includes three aspects: resistance, resilience, and adaptability [20,21,22]. It covers the whole process from food production, harvesting, processing, storage, and transportation to sales and consumption, involving multiple links.

### 2.2. Mechanisms of Influence

This paper combines the theoretical connotation of the food industry supply chain resilience and refers to many existing studies to explain how climate change affects the theoretical mechanism of the resilience of the food industry supply chain. First, on the economic side, the frequent occurrence of extreme weather events caused by climate change, such as droughts, floods, heat waves, etc., will directly damage the growth of crops, resulting in unstable and declining food production, and the reduction in food production will lead to insufficient supply, which in turn triggers an increase in food prices. Agriculture is one of the industries that are most directly affected by climate change, and the changes in temperature, precipitation, and other climatic factors profoundly affect crop production and food security [23]. The environmental aspect of the process of climate change must be considered, in which rising temperatures and changes in precipitation patterns as a result of climate change can alter the living environment of organisms, leading to the loss or fragmentation of the habitats of some species, which in turn affects the biodiversity of food production [11]. In the context of climate change and increasing urbanization, natural climate change and human activities are acting together in environmental change processes and forming synergistic forces [24]. Thirdly, on the social front, climate-change-induced food shortages and livelihood difficulties may lead to rural-urban or other areas of migration, and large-scale population movements may trigger social instability and competition for resources. Fourthly, on the policy front, support for agricultural research has been increased to promote the selection and breeding of resilient varieties, water-saving irrigation technology, smart agricultural technology and other research and development and applications. Meanwhile, from the theoretical basis and realistic logic, fiscal policy has become an indispensable component of public policy to address climate change [25]. Fifth, with regard to trade, over time, some regions of China have seen a decrease in the volume of food trade as a result of warmer temperatures. The instability of food production due to climate change has triggered sharp fluctuations in food prices on the international market, and the instability of international food prices thus increases the risk of food trade, which in turn affects the stability of the food market [26,27]. As the food production capacity of different regions is affected to varying degrees, global food trade flows may change, with some countries that were originally net exporters of food becoming net importers, while some countries with relatively favourable climatic conditions may increase the volume of their food exports.

**Figure 2:**
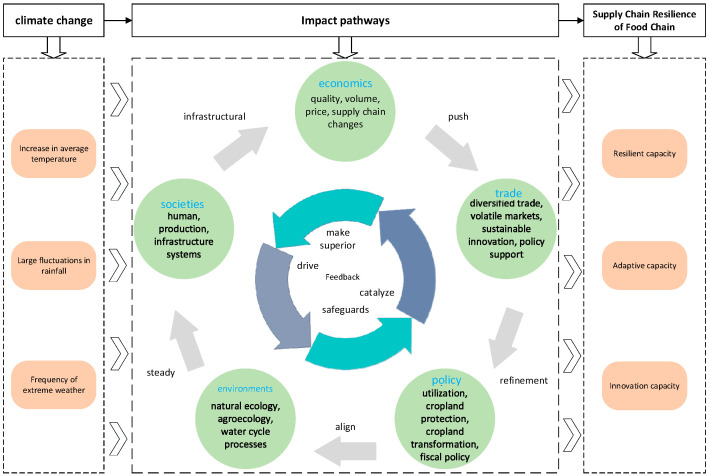
Impact mechanisms map.

This study synthesizes and maps the influencing mechanisms, as shown in detail in Figure 2.

### 2.3. Research Hypothesis

Climate change is a process that impairs the development of plant bodies by altering temperature and precipitation patterns, thereby affecting the thermal and hydrological conditions for plant growth, and this process of impact can lead to a reduction in food crop yields, especially in the case of extreme weather events, such as droughts, floods, heatwaves, hurricanes, etc., in which the direct loss of food yields is particularly pronounced [2]. At the same time, extreme weather also causes damage to transportation, logistics, energy, communications and other infrastructures, inhibiting the smooth flow of the food industry supply chain, reducing the efficiency of food logistics, and this impact will lead to various problems in the food distribution and sales chain, which will have an impact on the food trade market; at the same time, climate change will also lead to changes in the food supply and thus cause the price fluctuations of the world’s food market, and some countries have adopted export restrictions, agricultural support and other related policies to ensure the stability of the domestic food supply and price stability [28]. In order to ensure the stability of domestic food supply and price stability and take export restrictions, agricultural support and other related policies, this way will disrupt the international agricultural trade market, exacerbating the problem of food security in countries with a high degree of dependence on agricultural imports. Of course, climate change may also increase the level of risk in the food industry supply chain for a variety of reasons, including, but not limited to, disruptions in the food industry supply chain due to extreme weather events, difficulties in accessing food and agricultural resources, fluctuations in the prices of food raw materials and a decline in the quality of food products. These risks may have different impacts on all parts of the supply chain, and effective risk management strategies are needed to cope with such risks. The food industry supply chain also needs to adapt to the challenges posed by climate change, including improving resilience to extreme weather events, optimizing the allocation of food resources, and improving the flexibility of the food industry supply chain, which may involve redesigning the food industry supply chain and adjusting its operational strategies to improve its resilience to climate change [29,30]. In order to cope with the impacts of climate change, the food industry supply chain also requires continuous innovation and the adoption of new agricultural technologies and agricultural management practices, which can improve the efficiency of food production and the sustainability of food production. This includes the adoption of advanced agricultural technologies, improved supply chain management tools and sustainable agricultural practices. Food industry supply chains in different regions are affected by climate change to varying degrees, depending on local climatic conditions, agricultural production patterns and supply chain structures, and therefore require customized analyses of food industry supply chain in different regions.

Combined with the research on related climate change and the theoretical connotation of the food industry supply chain resilience, this paper proposes the following research hypotheses:

**H1:**  *Temperature changes negatively affect food industry supply chain resilience.*

**H2:**  *Rainfall variability negatively affect food industry supply chain resilience.*

In the context of climate change, food industry supply chain resilience will face unprecedented challenges. Extreme weather events caused by changes in temperature and rainfall will not only directly affect crop growth and crop yields, but also damage agricultural infrastructure and, to a certain extent, the marketing side of the food industry, thereby weakening the food industry supply chain resilience. In order to address these challenges, countries have adopted a series of adaptation measures, and crop diversification has been recognized as an effective strategy [31]. Crop diversification can improve resilience in various ways, such as by providing the ability to suppress pest outbreaks and dampen pathogen transmission, which may worsen under future climate scenarios, and by shielding crop production from the effects of climate variability and extreme events [32]. Different crops exhibit distinct responses to climatic factors. Drought-tolerant crops maintain baseline productivity during dry spells, and flood-tolerant crops ensure biomass accumulation during rainy periods. This asynchronous response mitigates the overall risk of climate shocks. Furthermore, crop diversification enhances the water-holding capacity and erosion resistance of farmland ecosystems by optimizing root-zone microclimates and improving soil structure, thereby reducing the direct effects of extreme precipitation or prolonged drought. From a supply chain perspective, crop diversification enables risk dispersion and balanced resource allocation. The differing phenological stages of various crops allow for staggered agricultural operations, avoiding the strain on labor, equipment, and irrigation resources caused by concentrated planting of a single variety. A diversified output structure also helps stabilize market price fluctuations. When one crop experiences reduced yields because of climatic factors, the profits from other crops can create a compensatory effect, maintaining the income stability of farming entities. On this basis, the following research hypothesis is proposed.

**H3:**  *Crop diversification can mitigate the negative effects of climate change on the resilience of food industry supply chains.*

### 2.4. Accelerated Genetic Algorithm Projective Tracer Modeling

In order to more accurately assess the impact of climate change on the food industry supply chain resilience, this paper adopts the projected tracer model of accelerated genetic algorithm (RAGA-PPC) instead of the traditional entropy method to construct the resilience index evaluation system of the food industry supply chain. RAGA-PPC is an optimization and evaluation model that combines the ideas of RAGA and PPC [20]. Currently, most of the related studies use traditional methods such as entropy value method to construct the food industry supply chain resilience index system, but these methods have certain limitations in dealing with complex variable relationships, because although the entropy value method is able to objectively determine the weights based on the degree of data discretization, it may not be able to adequately capture the intrinsic structure and characteristics of the data when confronted with the data that are high-dimensional, nonlinear, and have uncertainties.

In contrast, the Accelerated Genetic Algorithm Projection Tracing Model (RAGA-PPC) has significant advantages and is more suitable for the research needs of this paper. According to the study, the food industry supply chain resilience is affected by a variety of factors, which is difficult to be accurately portrayed by a simple linear model, and the RAGA-PPC model can optimize the optimal projection direction of high-dimensional data, map the complex multi-dimensional data into a one-dimensional space, so as to effectively reduce the dimensionality of the data, and exclude the interference of variables that have nothing to do with the data structure and features [21]. This method can not only successfully overcome the “curse of dimensionality” problem of high-dimensional data, but also open the way to solve high-dimensional problems using one-dimensional statistical methods. In summary, RAGA-PPC provides a more flexible, accurate and robust analytical framework for the study of the impact of climate change on the food industry supply chain resilience, which can effectively overcome the limitations of the traditional entropy value method and better adapt to the analysis of complex systems. The main steps are as follows:

Step 1: Create a collection of evaluation samples and normalize them to eliminate the effect of magnitude.

Step 2: Construct projection objective function Q(a).

Suppose that vector $a=(a_{1},a_{2},\ldots ,a_{n})$ is the projection direction. Then, the projection value Z(i) of the ith sample is

(1) $$Z\left(i\right)=\sum_{i=1}^{n}a_{j}*X^{*}\left(i,j\right),\;\;\;\;i=1,2,3,\ldots ,m$$

The expression of projection indicator function Q(a) is

(2) $$Q\left(a\right)\;=\;S_{z}\cdot D_{z},$$

where $S_{z}$ and $D_{z}$ are the standard deviation and local density of projected value $Z\left(i\right)$, respectively, with the following expressions:

(3) $$S_{z}\;=\;\sqrt{\frac{\sum_{i=1}^{n}{\left[Z\left(i\right)-E(z)\right]}^{2}}{n-1}},$$

(4) $$D_{z}=\sum_{i=1}^{n}\sum_{j=1}^{n}\left[R-r(i,j)\right]\cdot u[(R-r(i,j))],$$

where E(z) is the expected value of the projection; R is the radius of the window of local density, which can be determined by several trials;  r(i,j) represents the distance between the samples; and $\;u(R-r\left(i,j\right))$ is the unit leap function.

Step 3: Optimize the projection metric function. The optimal projection direction is to take the projection direction that maximizes the possibility of exposing a certain type of feature structure of the high-dimensional data. In this study, a real number coding-based accelerated genetic algorithm that simulates the mechanism of biological eugenics and chromosome information exchange within the population is applied to solve the high-dimensional global optimization problem. The optimal projection value is calculated as $a^{*}$. Equation (2) yields the maximization objective function

(5) $$MaxQ\left(a\right)\;=\;S_{z}\cdot D_{z},$$

(6) $$s.t.\sum_{j=1}^{p}a^{2}(j)=1,a\in [0,1].$$

Step 4: Analyze the composite evaluation index. Bringing $a^{*}$ obtained in Step 3 into Equation (1) yields the projected values for each sample province $Z^{*}(i)$, that is, the supply chain resilience evaluation index of the food industry supply chain in each province of the country.

### 2.5. Double Machine Learning Models

In order to examine the impact of climate change on the food industry supply chain resilience, this paper uses the Double Machine Learning (DML) model proposed by Chernozhukov et al. (2018) for causality identification [22]. Currently, most of the related studies use traditional methods to study causality, but most of these methods rely on a strict system of assumptions and usually require the assumption that there is a linear relationship between the variables; however, there are large limitations in the practical application of such assumptions. In contrast, double machine learning model has unique advantages in causal inference and is more suitable for the research problem of this paper.

On the one hand, as mentioned above, the food industry supply chain resilience will be affected by a combination of factors, including but not limited to agricultural production conditions, the volatility of the food market, the agricultural policy environment and other factors, which are intertwined with each other, and it is difficult to be accurately portrayed by a simple linear model. The double machine learning model can filter out the variables with higher prediction accuracy among a large number of high-dimensional control variables by machine learning algorithm, thus effectively controlling the interference of these complex factors on the food industry supply chain resilience, and avoiding the impact of the “curse of dimensionality” and multicollinearity on the estimation accuracy. On the other hand, at the micro level, the relationship between variables is often non-strictly linear, and the traditional linear regression model estimation method may have model setting bias, which leads to the estimation results are not robust enough. By contrast, the double machine learning model relaxes the linear assumptions between variables and can successfully capture nonlinear relationships and complex interaction effects, thus effectively avoiding model setting bias and ensuring the accuracy of causal relationship estimation between variables [22,33]. Therefore, the double machine learning model provides a more flexible, accurate and robust analytical framework for studying the impact of climate change on the food industry supply chain resilience.

Based on this, this paper constructs a double machine learning model to assess the impact of climate change on the food industry supply chain resilience as follows:

(7) $${FASCR}_{it}\;=\;\theta_{0}{GCC}_{it}+g(X_{it})+U_{it},$$

(8) $$E(U_{it}\;|\;{GCC}_{it},X_{it})=\;0,$$

where ${FASCR}_{it}$ denotes the level of resilience of the food industry supply chain, ${GCC}_{it}$ is the level of climate change, $X_{it}$ represents various control variables, $U_{it}$ is the random error term, $\theta_{0}$ refers to the climate change coefficient, and i and t denote the individual and year, respectively.

The direct application of machine learning algorithms to the estimation of the models in Equations (7) and (8) can easily lead to bias in the estimated $\hat{\theta }$, which in turn triggers estimation errors because of function regularity bias. To alleviate this problem, this study constructs an auxiliary regression model as follows:

(9) $${GCC}_{it}=m(X_{it})+V_{it},$$

(10) $$E\left(V_{it}|X_{it}\right)=0,$$

where $m(X_{it})$ is the regression function of the disposition variable in the high-dimensional control variable and $V_{it}$ is the error term with a conditional expectation of 0. A machine learning algorithm is used to estimate its specific form $\hat{m}(X_{it})$, and the estimate of its residuals is computed as ${\hat{V}}_{it}={GCC}_{it}-\hat{m}(X_{it})$. In addition, the machine learning algorithm is used to estimate the specific functional form $\hat{g}(X_{it})$ of $g(X_{it})$ in Equation (7). At this point, the functional form of the main regression changes to ${FASCR}_{it}-\hat{g}(X_{it})=\theta_{0}{GCC}_{it}+U_{it}$, and ${\hat{V}}_{it}$ is regressed as an instrumental variable in the explanatory variables ${GCC}_{it}$. The following unbiased estimates are obtained.

(11) $${\hat{\theta }}_{0}\;=\;(\frac{1}{n}\sum_{i\epsilon I,t\epsilon T}{\hat{V}}_{it}{GCC}_{it}{)}^{-1}\frac{1}{n}\sum_{i\epsilon I,t\epsilon T}{\hat{V}}_{it}\left\{{FASCR}_{it}-\hat{g}(X_{it})\right\}$$

For the DML model, this study employs random forest (RF) as the baseline model, and NNET is used for robustness testing. RF is configured as follows: 100 decision trees, unconstrained tree depth, random selection of features at each split equal to the square root of the total number of features, and a minimum sample size of 1 for leaf nodes. The neural network employs a three-layer architecture with 100 hidden layer units. The hidden layer uses the ReLU activation function, and the output layer employs a linear activation function. The optimizer is Adam with a learning rate of 0.001, trained for 100 iterations with an L2 regularization penalty coefficient of λ = 0.0001. All estimations are implemented in Stata 18.0, and pystacked 1.2.3 is used to call Python 3.9.7. RF relies on scikit-learn 1.0.2, and the neural network adopts TensorFlow 2.8.0. Cross-validation employs fivefold stratified sampling, which is repeated 101 times to enhance result stability.

### 2.6. Indicator System for Food Industry Supply Chain Resilience

The resilience of the food industry supply chain refers to the critical capacity of the food system to rapidly recover from external shocks and pressures, ensuring timely access to nutritious and healthy food for all individuals while maintaining the economic viability of all participants within the food system [11,12,13]. This study adopts a complex adaptive system theory framework and regards the food industry supply chain as a multilevel system characterized by dynamic feedback and self-organization. Its resilience manifests not only in passive shock resistance but also in internal regulatory adaptation and proactive transformative innovation capabilities. Building upon this framework and drawing from approaches proposed by relevant scholars [11,12,13,34], this study employs accelerated genetic algorithm projection pursuit to systematically construct a comprehensive evaluation index system for food industry supply chain resilience across three dimensions: resistance capacity, adaptation capacity, and innovation capacity. The details are presented in Table 1.

Resilience refers to the capacity of the food industry supply chain to mitigate and absorb risks when confronted with various internal and external threats and shocks. Its core lies in the robustness of resource foundations and production conditions. Therefore, indicators, such as food crop planting area, effectively irrigated land area, and agricultural land productivity, are selected to characterize the scale and quality of natural resources and land productivity, forming the material foundation of industry supply chain resilience. Per capita GDP and agricultural labor productivity are chosen to represent the support provided by economic and human capital to production stability. The agricultural producer price index is adopted as a negative indicator to depict the pressure that market fluctuations exert on system stability. The proportion of the workforce engaged in primary industries and per capita road area are employed to characterize the system’s foundational resilience.

Adaptability refers to the capacity of the food industry supply chain to maintain stability and recover to pre-shock operational states—or even achieve optimization and upgrading—through internal adjustments and adaptations when facing internal and external shocks and pressures. This capability focuses on enhancing resource utilization efficiency and risk resilience. Therefore, we select pesticide application per unit area, fertilizer application per unit area, and plastic film usage per unit area to represent the intensive utilization of agricultural inputs. Their reduction signifies the production system’s adaptive optimization in response to ecological and environmental pressures. Rural electricity consumption and rural broadband subscribers represent the support capacity of energy and information infrastructure for system regulation. The agricultural gross output growth index signifies the system’s vitality in output recovery, and the ratio of the disaster-affected area to the total affected area characterizes the system’s buffering effect against natural disasters.

Innovation capacity is a multidimensional concept encompassing organizational forms, information technology application, business model innovation, technological and industrial innovation, green transformation, and international trade. As a key driver for enhancing resilience and sustainability, it is measured by agricultural, forestry, animal husbandry, and fishery gross output value and per capita fixed asset investment in these sectors, which represent industrial scale and capital accumulation supporting innovation activities, and average power level, which reflects equipment and technological progress driving production efficiency. The amount of scientific research investment in the food industry and the number of R&D personnel are selected to measure the intensity of resource allocation for technological innovation. The import–export dependency ratio is adopted to reflect the open innovation strategy of enhancing market adaptability through international division of labor and cooperation.

### 2.7. Setting and Selection of Variables

#### 2.7.1. Explanatory Variables

The food industry supply chain resilience (FASCR), measured based on the accelerated genetic projection tracing model, is available.

#### 2.7.2. Core Explanatory Variables

Climate change is the central explanatory variable of this paper. Climate change refers to long-term changes in global or local climate states due to natural causes or human activities. Such changes may be characterized by long-term trends or statistically significant changes in temperature, precipitation, wind patterns, and other climate variables [35,36]. Climate change can have far-reaching effects on economic activities and social structures, including impacts on infrastructure, energy supply, and employment of the population. At the same time, climate change can also affect crop growth cycles and yields, which in turn can lead to food security issues. In this paper, referring to the practice of related scholars [37], the provincial average annual temperature (TEMP) and average annual rainfall (RAIN) were chosen to represent climate change from 2011 to 2022.

#### 2.7.3. Control Variable

To mitigate endogeneity and comprehensively control confounding factors, this study integrates the full-chain characteristics of the food industry supply chain (i.e., production, distribution, and macro-level safeguards) and references existing research. Seven categories of control variables are selected [38,39,40,41,42], each of which is grounded on explicit theoretical and empirical logic. Urbanization level (UL) reflects the effect of population and consumption structure on supply chain layout. The ratio of tertiary sector output to secondary sector output (RTSI) indicates the support of productive services for supply chain flexibility. Expenditures for the purchase of productive fixed assets (EPA) measures the risk resilience of infrastructure and production capacity. Energy efficiency (EUE) correlates with the stability of supply chain operational costs. The value of the output of specialized and auxiliary activities in agriculture, forestry, and fisheries (VAFA) represents the agricultural service system’s role in safeguarding the production end. Meanwhile, the ratio of the erosion control area to the total area (RECA) reflects the foundational influence of arable land quality on production resilience. Government general public budget expenditure as a ratio of GDP (RGPBE) reflects the macrolevel support of fiscal policies for supply chains. Together, these variables comprehensively cover key influence dimensions across the entire chain to precisely identify the net effects of climate change.

#### 2.7.4. Moderator Variable

In this study, crop diversification (measured by Simpson’s diversification index or SID) is selected as a moderating variable. Crop diversification has a statistically significant moderating effect on the resilience of the food industry supply chain. In the context of global warming and frequent occurrence of extreme weather, climate change exerts a serious effect on the resilience of the food industry supply chain. Crop diversification, as an important regulating variable, can effectively mitigate the negative influence of climate change on the resilience of the food industry supply chain [31]. Specifically, crop diversification can spread the risk of agricultural cultivation by increasing the types of agricultural cultivation and optimizing its structure, thus reducing the dependence on a single crop. When a crop is affected by climatic disasters or market fluctuations, other crops can play a buffer role and reduce the effect on the entire food industry supply chain. In addition, crop diversification can improve land utilization and resource use efficiency and enhance the stability of the agroecosystem. This diversified planting pattern can adapt to different climatic conditions and soil characteristics, giving full play to the advantages of regional planting and further enhancing the resilience of the food industry supply chain. In reality, due to the interference of various factors, the mitigating effect of crop diversity is absent and even exacerbates the influence of climate change on the resilience of the food industry supply chain. Thus, exploring the mitigating or exacerbating role of crop diversity in the effect of climate change on the resilience of the food industry supply chain is essential.

In this study, with reference to the conclusions of scholars at home and abroad, SID is used to measure the degree of crop diversification in food cultivation [31,43]. The specific calculation process is as follows.

Six types of crops, namely, rice, wheat, maize, beans, potatoes, and oilseeds, are selected in full consideration of the share of each type of crop to be grown. Indicator SID calculation proceeds as $\;SID=1-\sum N^{2}.{\;N}^{i}$ indicates the share of crop i in the total planted area. SID is bound by a scale of 0 to 1; 0 indicates complete specialization, and 1 indicates complete diversification. The value of SID provides a visual interpretation of crop diversity. The results of SID, which vary between 0 and 1, offer a visual representation of the level of diversity in food cultivation. The closer it is to 1, the more evenly distributed the crop is and the higher the level of diversity is.

### 2.8. Data Sources and Descriptive Statistical Analysis

Considering the completeness and availability of data, this paper excludes the Tibet Autonomous Region and Hong Kong, Macao and Taiwan, and selects the panel data of 30 provinces in China from 2011 to 2022 as the sample data to explore the impact of climate change on the resilience of the food industry supply chain. The agriculture-related indicators in this paper come from China Statistical Yearbook, China Science and Technology Statistical Yearbook, China Rural Statistical Yearbook, China Population and Employment Statistical Yearbook, China Agricultural Machinery Industry Yearbook, China Agricultural Yearbook, as well as provincial statistical yearbooks, China National Bureau of Statistics, etc.; and the meteorology-related indicators come from the National Meteorological Information Center’s National Meteorological Scientific Data Sharing Service Platform, etc. For some of the missing data, this paper adopts the linear interpolation method to complete. The descriptive statistics and definition results of each variable are shown in Table 2.

## 3. Results

### 3.1. Impact of Climate Change on the Resilience of the Food Industry Supply Chain

In order to empirically study the impact of climate change on the resilience of the food industry supply chain, the article adopts the random forest model of double machine learning, cuts the sample ratio to 1:4, and carries out cross-meshing so as to carry out the empirical analysis, and the specific results of the analysis are shown in Table 3. Columns (1) and (2) of Table 3 indicate the empirical results of the time effect and the individual effect are not fixed, and the results indicate that in the case of the time effect and the individual effect are not The results indicate that in the case of both time effect and individual effect are not fixed, the change in temperature has a negative effect on the resilience of the food industry supply chain, the size of the coefficient is −0.015, and it is significant at the 1% level of significance; the change in rainfall has a negative effect on the resilience of the food industry supply chain, the size of the coefficient is −0.001, and it is significant at the 1% level of significance. Columns (3) and (4) represent the regression results with only fixed time effects, and the results indicate that with only fixed time effects, both temperature and rainfall have a negative effect on the resilience of the food industry supply chain at the 1% level of significance, with coefficient sizes of −0.015, and −0.001, respectively. columns (5) and (6) represent the regression results with only fixed individual time effects, and the results indicate that with only individual time effects, the change in temperature has a negative effect on the resilience of the food industry supply chain, and the size of the coefficient is −0.001, and it is significant at 1%. case, changes in temperature have a negative effect on the resilience of the food industry supply chain, the size of the coefficient is −0.011, and it is significant at the 5% level of significance; changes in rainfall have a negative effect on the resilience of the food industry supply chain, the size of the coefficient is −0.001, and it is significant at the 10% level of significance. Columns (7) and (8) represent the empirical results with fixed time and individual effects, and the results indicate that with fixed time and individual effects, changes in temperature and rainfall have a negative impact on the resilience of the food industry supply chain at the 5% level of significance, with coefficients of −0.012 and −0.001, respectively, and it is clear from the results of the empirical analyses that, regardless of whether or not to take into account the time and individual effects, the temperature change and rainfall change have a negative impact on the resilience of the food industry supply chain, with coefficients of −0.001, respectively. From the results of empirical analysis, it can be seen that, regardless of whether the time effect and individual effect are considered or not, the changes in temperature and rainfall have a significant negative impact on the resilience of the food industry supply chain, and hypotheses H1 and H2 have been verified. This is consistent with the findings of existing studies on the impact of climate change on food systems [44].

### 3.2. Robustness Check

#### 3.2.1. Adjustment of the Study Sample

This paper takes into account the high level of urbanization in Shanghai and Chongqing, where food production, consumption and distribution are significantly affected by the urbanization process. And in the process of urbanization, a large amount of arable land has been occupied, leading to a reduction in the area under food cultivation. At the same time, urbanization also leads to the dietary and consumption habits of residents, increasing the reliance on processed food and eating out, etc. All factors may lead to interference with the overall results of the study on the resilience of the food industry supply chain, so this paper chooses to exclude the sample data of Shanghai and Chongqing in order to more accurately reflect the impact of climate change on the resilience of the food industry supply chain. The analysis results after the exclusion are shown in Table 4, in which columns (1) and (2) are the regression results of adjusting the research samples, and the results indicate that after the exclusion of the sample data of Shanghai and Chongqing, the change in temperature has a negative impact on the resilience of the food industry supply chain, with the coefficient size of −0.011, and it is significant at the 5% level of significance; and the change in rainfall has a negative impact on the resilience of the food industry supply chain with the coefficient size of −0.001, and significant at the 5% level of significance. It is easy to see that the empirical results are still robust compared with the previous regression results, which again verifies hypotheses H1 and H2.

#### 3.2.2. Removal of Outliers

In regression analysis, the outliers in the sample may lead to biased estimation results, and the outliers in the sample data will distort the model fitting effect, which will lead to inaccurate estimation of regression coefficients, and the error of the regression model will increase, which will reduce the reliability and interpretability of the regression results, this paper refers to the practice of Zhang and Li [45], and the explanatory variables, the explained variables, and the control variables in the model are subjected to shrinking treatment of 1%, 99% quartile, and 5%, 95% quartile, so as to carry out empirical analysis again, the empirical results are shown in the following table. 99% quantile and 5%, 95% quantile of the shrinkage treatment, will be higher than the highest quantile and lower than the lowest quantile of the value of the variables for alternative processing, so that the empirical analysis again, empirical results are shown in Table 4.

Table 4, columns (3) and (4) is the regression results of the data 1%, 99% shrinkage treatment, the results indicate that the temperature change and rainfall change are both at the 5% level of significance on the food industry supply chain resilience is negatively affected, the size of the coefficient is −0.011, −0.001, respectively, columns (5) and (6) for the data will be 5%, 95% shrink-tailed regression results, the results indicate that the temperature change in the 1% significant level on the food industry supply chain resilience is negatively affected, the size of the coefficient is −0.012, respectively; rainfall changes in the 5% significant level on the supply chain resilience of the food industry supply chain is negatively affected at the 5% significant level, and the size of the coefficient is −0.001; thus, it can be seen that the regression results are still robust after removing the outliers, and climate change still negatively affects the resilience of the food industry supply chain.

#### 3.2.3. Considering Time–Location Interaction Fixed Effects

In this paper, we introduce time-location interaction fixed effects (TLIFE) for robustness testing to control potential omitted variables and non-random selection bias, so as to ensure the robustness of the model results and the reliability of the model results. Meanwhile, since provinces are the key administrative units in China’s administrative governance structure, and cities within the same province usually show high similarity in terms of policy environment, location characteristics, and history and culture, this paper adds the time-location interaction fixed effects to the double machine learning model, and the empirical results are shown in Table 5. The regression results of fixed effects in Table 5 indicate that both temperature change and rainfall change negatively affect the resilience of the food industry supply chain at the 5% significant level, with coefficient sizes of −0.012 and −0.001, respectively, which further systematically proves the conclusions of the previous paper.

#### 3.2.4. Changing the Sample Cut Scale for Double Machine Learning Models

In double machine learning model, as the sample cutting ratio is critical to the training and validation process of the model. It will directly affect the estimation results of the model. And in limited samples, the division of specific subsamples is critical to the estimation of the results. By changing the sample cutting ratio, the model conclusion can be tested whether the model conclusion is sensitive to the sample division, so as to verify the robustness of the conclusion, so this paper will change the sample cutting ratio from 1:4 to 1:2 and 1:7, and then carry out the empirical analysis again, and the results of the analysis are shown in Table 5. Columns (9) and (10) in Table 5 show the regression results of changing the sample ratio to 1:2, and the results indicate that after replacing the sample cutting ratio of 1:2, the Both temperature change and rainfall change have a negative effect on the resilience of the food industry supply chain at a significant level of 5%, and the size of the coefficient is −0.013 and −0.001, respectively. Columns (11) and (12) in Table 5 are the regression results of changing the sample ratio to 1:7, and the results indicate that after replacing the sample cutting ratio of 1:7, the change in temperature has a negative effect on the resilience of the food industry supply chain, and the coefficient is −0.011, and the size is −0.011, and the size is −0.011, and the size is −0.011. size is −0.011 and significant at 5% significant level; rainfall changes have a negative effect on the resilience of the food industry supply chain, the coefficient size is −0.001 and significant at 10% significant level. The results show that changing the sample cutting ratio does not affect the conclusion that temperature change and rainfall change negatively affect the resilience of the food industry supply chain, which again verifies hypotheses H1 and H2.

#### 3.2.5. Replacement of Machine Learning Algorithms

This study examines the robustness of the validation by controlling for variables lagged by one period. First, we identify four endogenous control variables susceptible to climate shocks: VAFA, EPA, RGPBE, and EUE. These variables are incorporated into the DML model with one-period lag. The analysis results are presented in Columns (15) and (16) of Table 6. The findings indicate that after lagging the four control variables by one period, temperature changes exert a negative effect on the resilience of the food industry supply chain, with a coefficient magnitude of −0.014, which is statistically significant at the 5% level. Rainfall variability has a negative effect on the resilience of the food industry supply chain, with a coefficient magnitude of −0.001, which is statistically significant at the 10% level. The empirical results indicate that the influence of climate change on the resilience of the food industry supply chain remains robust and is unaffected by endogeneity or over-specification of control variables.

### 3.3. Heterogeneity Analysis

#### 3.3.1. Heterogeneity of Major Food-Producing Regions

In this paper, when investigating the impact of climate change on the resilience of the food industry supply chain, we take into account the significant heterogeneity between the main food-producing areas and non-food-producing areas in the impact trend. Meanwhile, with reference to the practice of other scholars [46], Chinese provinces are divided into main food-producing and non-food-producing areas. The main food-producing areas include 13 provinces, namely, Heilongjiang, Jilin, Liaoning, Inner Mongolia, Hebei, Henan, Shandong, Jiangsu, Anhui, Jiangxi, Hubei, Hunan, and Sichuan. The results of the specific heterogeneity analyses are shown in Table 7. As shown in (17) and (18) of Table 7, the major food-producing regions of the effects of temperature change and rainfall change on the resilience of the food industry supply chain are significantly enhanced, and the regression results are both significant at the 5% level of significance, while from columns (19) and (20) of Table 7, it can be seen that in the non-major food-producing regions, the effects of temperature change and rainfall change on the resilience of the food industry supply chain are not significant.

#### 3.3.2. Heterogeneity in the Level of Mechanization

In the empirical analysis examining the impact of climate change on the resilience of the food industry supply chain, the heterogeneity of mechanization levels cannot be ignored. Referring to the practice of other scholars [47], the ratio of the total power of agricultural machinery to the area of cultivated land is used to measure the regional level of agricultural mechanization, and the samples are divided into two groups of low mechanization and high mechanization according to the median of the mechanization level, and this heterogeneity has far-reaching impacts on the stability and resilience of the food industry supply chain, and therefore has an important research necessity. As can be seen from columns (21) and (22) in Table 8, in the high mechanization level group, temperature change has a negative impact on the resilience of the food industry supply chain at a significant level of 1%, with a coefficient size of −0.020; rainfall change has a negative impact on the resilience of the food industry supply chain at a significant level of 10%, with a coefficient size of −0.001; in comparison, in the low mechanization level group, the effects of temperature change and rainfall change on the resilience of the food industry supply chain are not significant.

### 3.4. Moderating Effect

Different crops have different adaptability to climate change, and crop diversification may reduce the fluctuation of food production due to climate anomalies. And when a crop is affected by climate change and production is reduced, other crops can be supplemented to ensure the stability of the industrial chain, which reduces the dependence on a single crop, thus improving the overall resilience [34]. In this paper, the impact of climate change on the resilience of the food industry supply chain regression model to add the moderating effect of crop diversification, the regression results are shown in Table 8. columns (25) and (16) in Table 9 are the regression results of the model after adding the crop diversification variable and the interaction term between crop diversification and explanatory variables. As shown in Column (26), the main effect coefficient of temperature variation on the resilience of the food industry supply chain is positive, with a regression coefficient of −0.148, and is significant at the 1% level. Compared with the model without the crop diversification variable, the direction of the impact remains unchanged, but the magnitude of the impact has significantly increased. Furthermore, both crop diversification and the interaction term between crop diversification and temperature variation are statistically significant at the 1% level. These findings indicate that crop diversification, acting as a moderator, substantially buffers the negative effect of temperature variation on the resilience of the food industry supply chain, thereby validating Hypothesis 3. However, in column (25), in the regression of the model after the addition of crop diversification, the regression results of the resilience of the food industry supply chain on the change in rainfall are missing and insignificant, and at the same time, the interaction terms of crop diversification and crop diversification with the change in rainfall are also insignificant, leading to the lack of significant results of the model without the addition of crop diversification variables, which leads to the lack of significant effects. The interaction terms of crop diversification and crop diversification with rainfall change are also not significant, which may be due to the complexity of the mechanism of rainfall change on the resilience of the food industry supply chain and the irregular changes in rainfall in recent years, which makes it difficult to play a stabilizing role for crop diversification.

## 4. Conclusions and Policy Implications

Based on the panel data of 30 provinces in China from 2011 to 2022, this paper empirically investigates the impact of climate change on the resilience of the food industry supply chain, and makes the analysis of the moderating effect and heterogeneity analysis on this basis. This study found that: first, climate change has a significant negative impact on the resilience of the food industry supply chain, and in climate change, the impact of temperature on the resilience of the food industry supply chain is significantly higher than the impact of rainfall on the resilience of the food industry supply chain, and the test results passed the robustness test. Second, the impact of climate change on the resilience of the food industry supply chain has obvious heterogeneity between the major food-producing regions and the non-major food-producing regions, in which the impact of temperature change and rainfall change on the industry supply chain resilience of the food chain in the major food-producing regions is significantly enhanced, while the impact of the non-major food-producing regions is not significant. Third, the impact of climate change on the resilience of the food industry supply chain varies across mechanization levels, with the impacts of temperature change and rainfall change on the resilience of the food industry supply chain in the main food-producing areas being significant in areas with high mechanization levels, but not in areas with low mechanization levels. Fourth, crop diversification substantially mitigates the negative effect of temperature fluctuations on the resilience of the food industry supply chain.

Based on the above findings, this paper makes the following policy recommendations.

First, the climate resilience and disaster resistance of food industry supply chains should be systematically enhanced. Their climate adaptation and disaster resilience must be systematically strengthened to directly counter the negative effects of climate change. This task includes promoting the research, development, and adoption of adaptive crop varieties; supporting the breeding and commercial cultivation of heat-tolerant, drought-resistant, and pest-resistant seeds to reduce the influence of climate fluctuations on crop yields; establishing a precision early-warning system for agricultural meteorological disasters; optimizing the layout of monitoring networks; and creating a climate risk early-warning mechanism that covers multiple stages, including production, storage, and distribution, to enhance rapid response capabilities across the entire chain.

Second, differentiated regional climate adaptation strategies should be implemented, and policies and resources for major food-producing regions should be prioritized. Given that climate change exerts a strong effect on the resilience of food industry supply chains in these areas, differentiated regional climate adaptation strategies should be formulated and implemented to enhance the overall national food security. This task requires prioritizing policies and resources for major food-producing regions. Specifically, investments in climate-smart agricultural infrastructure should be increased in these regions. High-standard drought-resistant and flood-proof facilities should be constructed, precision irrigation technologies should be promoted, and agricultural disaster monitoring and early warning systems must be deployed. Concurrently, a dedicated climate resilience fund should be established for major food-producing areas to compensate for the additional climate risks that they bear in safeguarding national food security. The development of financial instruments, such as climate index insurance, should also be encouraged to mitigate systemic risks. For non-food-producing regions that are minimally affected by climate change, efforts should concentrate on cultivating their latent production capacity. This task can be achieved by enhancing agricultural technology dissemination, strengthening the routine maintenance of farmland infrastructure, and encouraging the development of specialty crops suited to local climates. Such regions could then serve as effective strategic supplements and buffer zones during extreme weather events that reduce yields in primary production areas, collectively enhancing the overall stability of the national food industry supply chain.

Third, for highly mechanized regions, the application of smart agricultural machinery and climate-adaptive technologies must be intensified. Research has found that climate change effects are highly pronounced in highly mechanized areas, reflecting their considerable dependence on and sensitivity to climatic factors. Consequently, efforts should concentrate on enhancing the climate resilience of agricultural machinery and operational systems. Such enhancement includes prioritizing the development of specialized machinery resistant to high temperatures and waterlogging and promoting AI-based precision climate monitoring and intelligent irrigation systems. By establishing an integrated sky–ground intelligent scheduling platform, real-time meteorological data can be obtained to dynamically optimize agricultural machinery operations, effectively mitigating extreme weather disruptions to mechanized processes and stabilizing production efficiency in core industry supply chain segments.

Fourth, for regions with low mechanization levels, efforts should concentrate on addressing shortcomings in agricultural machinery application and infrastructure. In these areas, the limited influence of climate change reflects insufficient mechanization and the underutilization of existing production capacity constrained by climate factors. Fundamental improvements in machinery penetration rates and application capabilities are required. Specific measures include increasing subsidies for agricultural machinery purchases, promoting small-to-medium-sized machinery suited to local topography and climate, organizing training in agricultural machinery operation under extreme weather conditions to enhance operator skills, and prioritizing improvements to infrastructure (e.g., farm roads and irrigation channels) to ensure that machinery that can access fields and water is readily available. Doing so will fundamentally strengthen these regions’ capacity to withstand climate risks.

Fifth, crop diversification should be systematically promoted to mitigate temperature fluctuation risks. Research has shown that crop diversity can buffer the negative effects of temperature changes on the resilience of the food industry supply chain. Therefore, high priority should be given to systematically promoting crop diversification to effectively address temperature variation risks. Specifically, this involves integrating crop diversification development into the national food security framework, formulating specialized development plans, and scientifically allocating crop types and varieties with differing climate adaptability across major agricultural regions. In addition, support for collecting and breeding stress-tolerant germplasm resources, such as heat- and drought-resistant varieties, should be strengthened. The large-scale adoption of ecological farming practices, such as intercropping and crop rotation, must be promoted while providing ecological compensation and technical subsidies to farmers who adopt diverse cropping systems. Concurrently, investment in supporting infrastructure should be enhanced by establishing intelligent farm management platforms to achieve precise alignment between climate risks and crop growth patterns.

## 5. Discussion

The resilience evaluation system for the food industry supply chain constructed in this study adopts a core three-dimensional framework of resistance capacity–adaptation capacity–innovation capacity. This approach aligns closely with international classical research. Tendall’s [48] three-dimensional deconstruction framework for resilience is the core theoretical basis of this study’s framework. Zuo Xiuping [13], Zheng Junchuan [11], and others adopted the three-dimensional resistance–adaptation–innovation framework as the theoretical foundation for resilience measurement, and they all conducted empirical analyses by using provincial-level panel data. Furthermore, this empirical study finds that temperature and rainfall variations considerably suppress the resilience of the food industry supply chain. Temperature changes exert a markedly stronger influence on resilience than rainfall variations, a finding that is consistent with those of existing research on climate change’s effects on food industry supply chain resilience [24,49]. Furthermore, this study empirically demonstrates that crop diversification mitigates the negative effect of temperature fluctuations on the resilience of the food industry supply chain, broadly aligning with the findings of Brenda [32].

This section briefly summarizes the key findings of this study and highlights its research value. But this paper still has certain limitations: first, this paper is only based on the analysis of the provincial level, and does not go deeper into the municipal level, county level for comprehensive research, the future should be deeper into the municipal level, the county level to explore the In the future, the impact mechanism of climate change on the resilience of the food industry chain supply chain should be explored at the municipal and county levels. Second, this paper only discusses the impact of climate change on the resilience of the food industry chain supply chain, but does not explore the counteraction mechanism of the food industry chain supply chain system on climate change, future research can start from the perspective of carbon emission and carbon sequestration of the food industry chain supply chain system to explore its impact on climate change. Third, future research should not only focus on the domestic perspective, but also strengthen strategic research on international and multi-regional food production security.

## Figures and Tables

**Table 1:** Indicator system for the food industry supply chain resilience.

Level 1 Indicators	Level 2 Indicators	Causality	Weights	Variable Missing Rate
Ability to resist of the food industry supply chain	Area sown in food crops	Positive	0.0545105	0%
	Effective irrigated area	Positive	0.0675145	0%
	GDP per capita	Positive	0.0397404	0%
	Food production per unit area	Positive	0.0499404	0%
	Agricultural land productivity	Positive	0.0413300	0%
	Agricultural labor productivity	Positive	0.0500949	0%
	Producer price index for agricultural products	Negative	0.0443326	0%
	Percentage of personnel in the primary sector	Positive	0.0254338	0%
	Road area per capita	Positive	0.0370248	9.9722%
Adaptability of the food industry supply chain	Pesticide application rate	Negative	0.0614872	8.3102%
	Chemical fertilizer application rate	Negative	0.0572261	0%
	Plastic film usage	Negative	0.0605378	0%
	Rural electricity consumption	Positive	0.0434053	0%
	Gross agricultural output growth index	Positive	0.0372676	0%
	Number of rural broadband access subscribers	Positive	0.0425222	1.9191%
	Area affected/area affected × 100%	Negative	0.0307105	0%
Innovation capability of the food industry supply chain	Gross output value of agriculture, forestry, livestock and fisheries	Positive	0.0625028	0%
	Per capita investment in fixed assets in agriculture, forestry, fisheries and livestock	Positive	0.0281273	0%
	Average power level	Positive	0.0391884	0%
	Amount of research investment in the food industry	Positive	0.0549300	0%
	Number of R&D personnel in the food industry	Positive	0.0490352	0%
	import–export dependence	Positive	0.0231377	0%

**Table 2:** Descriptive statistics.

Variable Type	Variable Name	Average Value	Standard Deviation	Maximum Values	Minimum Value	Description of Variables
Explained variable	FASCR	1.3185	0.4716	2.7035	0.5299	Food industry supply chain resilience
Explanatory variable	TEMP	76.9834	40.4430	178.4840	8.2231	Average annual temperature
	RAIN	12.7349	6.1196	25.2346	−2.8507	Average annual rainfall
Moderator variable	SID	0.7992	0.1047	0.9429	0.4619	Crop diversity index
	UL	0.6014	0.1205	0.8958	0.3503	Urbanization level
	RTSI	1.2658	0.7218	5.3101	0.5183	Ratio of tertiary sector output to secondary sector output
	EPA	320.4596	327.0145	2317.7200	5.3000	Expenditures on the purchase of productive fixed assets
	EUE	0.7946	0.4814	2.3271	0.2076	Energy efficiency
	VAFA	195.6431	183.0225	963.7000	4.7600	Value of the output of specialized and auxiliary activities in agriculture, forestry, and fisheries
	RECA	0.2199	0.1439	0.6040	0.0000	Ratio of erosion control area to total area
	RGPBE	0.2590	0.1115	0.7583	0.1050	Government general public budget expenditure as a ratio of GDP

**Table 3:** Double machine learning regression results.

Variable	(1)	(2)	(3)	(4)	(5)	(6)	(7)	(8)
	FASCR	FASCR	FASCR	FASCR	FASCR	FASCR	FASCR	FASCR
RAIN	−0.001 *** (−2.98)		−0.001 *** (−3.25)		−0.001 * (−1.84)		−0.001 ** (−1.99)	
TEMP		−0.015 *** (−3.63)		−0.015 *** (−3.63)		−0.011 ** (−2.41)		−0.012 ** (−2.36)
Control variable	YES	YES	YES	YES	YES	YES	YES	YES
Cross fitting	5	5	5	5	5	5	5	5
Time fixed	NO	NO	YES	YES	NO	NO	YES	YES
Individual fixed	NO	NO	NO	NO	YES	YES	YES	YES
Sample size	360	360	360	360	360	360	360	360

*, **, and *** mean passing a significance test at 10%, 5%, and 1% levels, respectively.

**Table 4:** Robustness Test 1 of Regression Results on the Impact of Climate Change on the Resilience of the Food Industry Supply Chain.

Variable	(1)	(2)	(3)	(4)	(5)	(6)
	Adjustment of the Study Sample	Adjustment of the Study Sample	1% Indentation	1% Indentation	5% Indentation	5% Indentation
	FASCR		FASCR		FASCR	
RAIN	−0.001 ** (−1.97)		−0.001 ** (−2.01)		−0.001 ** (−2.15)	
TEMP		−0.011 ** (−2.17)		−0.011 ** (−2.37)		−0.012 *** (−2.82)
Control variable	YES	YES	YES	YES	YES	YES
Cross fitting	5	5	5	5	5	5
Time fixed	YES	YES	YES	YES	YES	YES
Individual fixed	YES	YES	YES	YES	YES	YES
Sample size	336	336	360	360	360	360

*, **, and ***mean passing a significance test at 10%, 5%, and 1% levels, respectively.

**Table 5:** Robustness test 2 of Regression Results on the Impact of Climate Change on the Resilience of the Food Industry Supply Chain.

Variable	(7)	(8)	(9)	(10)	(11)	(12)	(13)	(14)
	TLIFE	TLIFE	Changing the Cutting Ratio	Changing the Cutting Ratio	Changing the Cutting Ratio	Changing the Cutting Ratio	Nnet Model	Nnet Model
	FASCR		FASCR		FASCR		FASCR	
RAIN	−0.001 ** (−2.15)		−0.001 ** (−2.38)		−0.001 * (−1.81)		0.002 ** (2.06)	
TEMP		−0.012 ** (−2.50)		−0.013 ** (−2.38)		−0.011 ** (−2.34)		0.012 * (1.76)
Control variable	YES	YES	YES	YES	YES	YES	YES	YES
Cross fitting	5	5	3	3	8	8	5	5
Time fixed	YES	YES	YES	YES	YES	YES	YES	YES
Individual fixed	YES	YES	YES	YES	YES	YES	YES	YES
Sample size	360	360	360	360	360	360	360	360

*, **, and *** mean passing a significance test at 10%, 5%, and 1% levels, respectively.

**Table 6:** Robustness test 3 of Regression Results on the Impact of Climate Change on the Resilience of the Food Industry Supply Chain.

Variable	(15)	(16)
	Control Variables Lagged by One Period	Control Variables Lagged by One Period
	FASCR	FASCR
RAIN	−0.001 * (−1.81)	
TEMP		−0.014 ** (−2.43)
Control variable	YES	YES
Cross-fitting	5	5
Time fixed	YES	YES
Individual fixed	YES	YES
Sample size	360	360

*, **, and *** mean passing a significance test at 10%, 5%, and 1% levels, respectively.

**Table 7:** Heterogeneity Analysis Results of the Impact of Climate Change on the Resilience of the Food Industry Supply Chain in Major Food-Producing Areas and Non-Major Food-Producing Areas.

Variable	(17)	(18)	(19)	(20)
	Major Food-Producing Regions		Non-Dominant Food-Producing Regions	
	FASCR	FASCR	FASCR	FASCR
RAIN	−0.002 ** (−2.36)		0.000 (0.23)	
TEMP		−0.019 ** (−1.97)		−0.001 (−0.26)
Control variable	YES	YES	YES	YES
Cross fitting	5	5	5	5
Time fixed	YES	YES	YES	YES
Individual fixed	YES	YES	YES	YES
Sample size	156	156	204	204

*, **, and *** mean passing a significance test at 10%, 5%, and 1% levels, respectively.

**Table 8:** Heterogeneity Analysis Results of the Impact of Climate Change on the Resilience of the Food Industry Supply Chain under Different Levels of Mechanization.

Variable	(21)	(22)	(23)	(24)
	High Mechanization		Low Mechanization	
	FASCR	FASCR	FASCR	FASCR
RAIN	−0.001 * (−1.77)		−0.001 (−1.22)	
TEMP		−0.020 *** (−2.67)		−0.009 (−1.11)
Control variable	YES	YES	YES	YES
Cross fitting	5	5	5	5
Time fixed	YES	YES	YES	YES
Individual fixed	YES	YES	YES	YES
Sample size	180	180	180	180

*, **, and *** mean passing a significance test at 10%, 5%, and 1% levels, respectively.

**Table 9:** Analysis Results on the Moderating Effect of Crop Diversity in the Impact of Climate Change on the Resilience of the Food Industry Supply Chain.

Variable	(25)	(26)
	Moderating Effect	Moderating Effect
	FASCR	FASCR
RAIN	−0.004 (−1.45)	
TEMP		−0.148 *** (−4.72)
Control variable	−0.107 (−0.34)	−1.703 *** (−4.22)
Cross fitting	0.005 (1.51)	0.167 *** (5.53)
Time fixed	YES	YES
Individual fixed	5	5
Sample size	YES	YES

*, **, and *** mean passing a significance test at 10%, 5%, and 1% levels, respectively.

## Data Availability

The original contributions presented in this study are included in the article. Further inquiries can be directed to the corresponding author.
